# Weight loss required by the severely obese to achieve clinically important differences in health-related quality of life: two-year prospective cohort study

**DOI:** 10.1186/s12916-014-0175-5

**Published:** 2014-10-15

**Authors:** Lindsey M Warkentin, Sumit R Majumdar, Jeffrey A Johnson, Calypse B Agborsangaya, Christian F Rueda-Clausen, Arya M Sharma, Scott W Klarenbach, Shahzeer Karmali, Daniel W Birch, Raj S Padwal

**Affiliations:** Department of Medicine, University of Alberta, Edmonton, AB Canada; Alberta Diabetes Institute, Edmonton, AB Canada; School of Public Health, University of Alberta, Edmonton, AB Canada; Department of Surgery and CAMIS (Center for the Advancement of Minimally. Invasive Surgery), University of Alberta, Royal Alexandra Hospital, Edmonton, AB Canada; 5-134A Clinical Sciences Building, 8440-112th Street, Edmonton, AB T6G 2G3 Canada

**Keywords:** Health-related quality of life, Weight loss, Minimal clinically important difference, Obesity, Patient reported outcomes, Bariatric care

## Abstract

**Background:**

Guidelines and experts describe 5% to 10% reductions in body weight as ‘clinically important’; however, it is not clear if 5% to 10% weight reductions correspond to clinically important improvements in health-related quality of life (HRQL). Our objective was to calculate the amount of weight loss required to attain established minimal clinically important differences (MCIDs) in HRQL, measured using three validated instruments.

**Methods:**

Data from the Alberta Population-based Prospective Evaluation of Quality of Life Outcomes and Economic Impact of Bariatric Surgery (APPLES) study, a population-based, prospective Canadian cohort including 150 wait-listed, 200 medically managed and 150 surgically treated patients were examined. Two-year changes in weight and HRQL measures (Short-Form (SF)-12 physical (PCS; MCID = 5) and mental (MCS; MCID = 5) component summary score, EQ-5D Index (MCID = 0.03) and Visual Analog Scale (VAS; MCID = 10), Impact of Weight on Quality of Life (IWQOL)-Lite total score (MCID = 12)) were calculated. Separate multivariable linear regression models were constructed within medically and surgically treated patients to determine if weight changes achieved HRQL MCIDs. Pooled analysis in all 500 patients was performed to estimate the weight reductions required to achieve the pre-defined MCID for each HRQL instrument.

**Results:**

Mean age was 43.7 (SD 9.6) years, 88% were women, 92% were white, and mean initial body mass index was 47.9 (SD 8.1) kg/m^2^. In surgically treated patients (two-year weight loss = 16%), HRQL MCIDs were reached for all instruments except the SF-12 MCS. In medically managed patients (two-year weight loss = 3%), MCIDs were attained in the EQ-index but not the other instruments. In all patients, percent weight reductions to achieve MCIDs were: 23% (95% confidence interval (CI): 17.5, 32.5) for PCS, 25% (17.5, 40.2) for MCS, 9% (6.2, 15.0) for EQ-Index, 23% (17.3, 36.1) for EQ-VAS, and 17% (14.1, 20.4) for IWQOL-Lite total score.

**Conclusions:**

Weight reductions to achieve MCIDs for most HRQL instruments are markedly higher than the conventional threshold of 5% to 10%. Surgical, but not medical treatment, consistently led to clinically important improvements in HRQL over two years.

**Trial registration:**

Clinicaltrials.gov NCT00850356.

**Electronic supplementary material:**

The online version of this article (doi:10.1186/s12916-014-0175-5) contains supplementary material, which is available to authorized users.

## Background

Class II (body mass index (BMI) 35 to 39.9 kg/m^2^) and Class III (BMI ≥40 kg/m^2^) obesity (hereafter collectively referred to as ‘severe’ obesity) have increased by 400% over two decades and lead to substantial morbidity, mortality and reduced health-related quality of life (HRQL) [1-4]. Contemporary guidelines define 5% to 10% weight reductions as clinically important, citing expert opinion and statistically significant (albeit modest) improvements in cardio-metabolic risk as evidence for this contention [5-8]. Although many studies have examined HRQL (perceptions of physical, mental and social functioning) changes following weight loss [9,10], to our knowledge, none have attempted to calculate the amount of weight loss required to achieve minimal clinically important differences (MCIDs) in HRQL or verify that 5% to 10% weight reductions result in clinically important HRQL improvements.

An MCID is the smallest difference in score in the domain of interest which patients perceive as beneficial and which would mandate a change in the patient’s management [11,12]. HRQL, unlike various cardio-metabolic parameters or weight, is not a surrogate or intermediate measure; rather, it is a patient-reported outcome of tremendous clinical importance in its own right [8]. With the expanded use of HRQL endpoints and the increasing number of HRQL instruments (each with its own scoring structure and scale), interpreting HRQL in the context of MCID improvements is imperative. Thus, empirically determining the weight reduction thresholds corresponding to these MCIDs is needed. These instrument-specific weight loss thresholds could then be used to assess whether new or existing treatments are producing clinically important HRQL improvements.

The objective of this study was to examine treatment-related HRQL change and define clinically important weight loss as it relates to HRQL (that is, to determine the weight reductions required to achieve HRQL MCIDs). Specifically, we used two-year longitudinal data from 500 severely obese patients enrolled in a population-representative bariatric program to determine: (1) the two-year changes in weight and HRQL with medical and surgical treatment; and (2) the amount of weight loss required to attain MCIDs for three validated HRQL instruments.

## Methods

A detailed study protocol for the Alberta Population-based Prospective Evaluation of the Quality of Life Outcomes and Economic Impact of Bariatric Surgery (APPLES) study, a prospective two-year observational evaluation of surgically treated, medically managed and wait-listed severely obese patients has been previously published [13]. The University of Alberta Health Research Ethics Board approved the study and all patients provided written informed consent.

### Participants

Patients enrolled in APPLES were recruited from the adult specialty clinic of the Edmonton Weight Wise regional obesity program. Weight Wise has a central, region-wide, single-point-of-access referral system for the 1.6 million residents of the Edmonton Zone of Alberta Health Services. The adult specialty clinic provides both medical and surgical treatment to practitioner-referred patients ≥18-years-old with BMI levels ≥35 kg/m^2^ who have been unsuccessful with prior attempts at managing chronic obesity. Importantly, patients sequentially progress through the program, from the wait-list to medical management and (if appropriate) to surgery (approximately 65% of medically managed patients eventually receive surgery). At the time APPLES was conducted, the mean entry wait time was two years.

APPLES was a naturalistic assessment of outcomes in the adult clinic of Weight Wise. Patients without surgical contraindications were enrolled. One hundred fifty patients approved for surgery, 200 patients initiating medical management and 150 patients wait-listed were consecutively enrolled between January 2009 and February 2010. Patients in each study group were enrolled just after they entered that particular phase of the program. Consistent with the ‘pragmatic’ nature of the study, no attempt was made to delay surgery if approval was obtained. The enrollment target was higher in the medical group to account for expected higher attrition (due to patients’ crossing over to surgery over the two-year period) [13,14]. Given the sequential nature of the program, surgically treated patients would have previously received medical management and both medical and surgical patients would have previously been wait-listed.

Wait-listed patients were advised to attend community-based group education sessions prior to clinic entry, but otherwise received no specific intervention. Medically-managed patients received at least 24 weeks of individualized, intensive, lifestyle counseling (diet, exercise, behavioral modification) based on contemporary Canadian obesity guidelines [5]; regarding physical activity, patients were provided individualized recommendations for how to increase physical activity, without any formal exercise program being initiated. Surgically-treated patients underwent Roux-en-Y gastric bypass, gastric banding, or sleeve gastrectomy [13,14].

### Measurements

Assessments were not blinded. Baseline data included age, sex, comorbidities, smoking status, medications, weight and cardio-metabolic parameters [13]. Body weight was measured as previously described [13] to the nearest 0.1 kg every six months for two years.

### HRQL measures

All patients completed the Short Form (SF)-12 Version 2, the EuroQoL-5 dimensions (EQ-5D), and Impact of Weight on Quality of Life (IWQOL)-Lite surveys at the time of entry into the cohort and every six months for two years. These three validated instruments were chosen to comprehensively assess HRQL outcomes, from generic to obese-specific [15,16]. The SF-12 is a condensed version of the SF-36, a commonly used generic health-status tool [17]. It yields a physical and a mental health component summary score, referred to as PCS and MCS, respectively, which follow a T distribution (mean 50, SD 10), normalized for the general US population. Higher scores indicate better health status. A three-to-five point increase in PCS or MCS score is considered clinically important [18,19]. Given the severe baseline HRQL impairment present in our population, and that larger improvements may be expected in individuals with lower baseline scores, we used a score of 5 as the MCID threshold [20].

The EQ-5D is a preference-based health survey assessing five health dimensions (with three levels of problems) and an overall health visual analog scale (EQ-VAS) [21]. The descriptive system is scored using a set of weights representing the general population’s preferences, into a single summary (EQ-index) anchored at 0 (death) and 1 (full health). The EQ-VAS score ranges from 0 (worst imaginable health state) to 100 (best imaginable health state). The established MCID for the EQ-index score is 0.03 points, while 10 points is the MCID for the EQ-VAS [22].

The IWQOL-Lite is used to assess obesity-specific HRQL [23]. It consists of 31 items describing 5 domains (physical function, self-esteem, sexual life, public distress and work). Total scores range from 0 to 100 (with lower scores indicating greater impairment), with an MCID of 7 to 12 [12]. We used the higher end of this range (12) as the MCID, as is recommended if baseline HRQL impairment is severe [24].

### Statistical analysis

Between-group baseline variables were compared using one-way analysis-of-variance (ANOVA) for continuous outcomes and chi-squared tests for dichotomous ones.

### HRQL changes with medical and surgical treatment

Within-group two-year changes in weight, BMI and all five HRQL scores were calculated. Mean wait-list subtracted improvements in HRQL for medically managed and surgically-treated patients were calculated for each instrument, and adjusted for age, sex, baseline BMI, and baseline HRQL score. These improvements were wait-list adjusted to control for temporal changes in HRQL not associated with specific treatment. Proportions of medically managed and surgically treated patients meeting the established HRQL MCID were calculated for each instrument (wait-listed proportions are also presented). Between-group differences in these proportions were analyzed using chi-square tests. *P*-values <0.05 were considered statistically significant.

### Weight loss required to attain HRQL MCIDs

In all 500 participants, instrument-specific multivariable linear regression models were constructed to determine the independent associations between two-year changes in weight and HRQL scores. Models were adjusted for age, sex and baseline BMI, HRQL and study arm. The weight change model coefficient was used to calculate the weight loss required to achieve HRQL MCIDs for each instrument.

In trying to conduct a modified ‘intent-to-treat’ analysis, patients were analyzed according to the group to which they were originally allocated. Thus, once patients transitioned from the wait-list to medical-management or from medical-management to surgery they stopped contributing data and were censored. As established *a priori*, last-observation-carried-forward (LOCF) imputation for both HRQL data and weight was used to account for data missing as a result of censoring or loss-to-follow up [13]. Multiple imputation was not performed because the data are not missing at random [25]. All analyses were performed using STATA (Version 13 SE, College Station, TX, USA).

## Results

### Baseline characteristics

Mean age was 43.7 (9.6) years, mean weight was 131.9 (25.1) kg, mean BMI was 47.9 (8.1) kg/m^2^, and 88% were female (Table 1). Body weight and BMI were significantly lower in the surgical group compared to the other groups (*P* = 0.05 for weight and *P* = 0.003 for BMI). Conversely, all HRQL scores were significantly higher in the surgical group compared to the other groups (*P* <0.001 for all comparisons).

**Table 1:** **Baseline characteristics**

**Characteristic**	**Wait-listed (number = 150)**	**Medical-management (number = 200)**	**Surgical-treatment (number = 150)**	***P*** **-value** ^**a**^
Female (number (%))	136 (91)	174 (87)	131 (87)	0.5
Age (years, mean (SD))	43.6 (9.2)	43.9 (10.0)	43.5 (9.5)	0.9
Married (number (%))	80 (54)	116 (58)	93 (62)	0.1
White (number (%))	139 (93)	178 (89)	141 (94)	0.2
Weight (kg, mean (SD))	134.7 (25.1)	132.9 (24.7)	127.9 (25.2)	0.05
BMI (kg/m2, mean (SD))	49.4 (8.2)	48.0 (8.2)	46.2 (7.4)	0.003
Hypertension (number (%))	99 (66)	134 (67)	92 (61)	0.5
Dyslipidemia (number (%))	89 (59)	123 (62)	90 (60)	0.2
Diabetes (number (%))	75 (50)	80 (40)	67 (45)	0.9
Depression (number (%))	98 (65)	133 (67)	88 (59)	0.3
SF-12 PCS (mean (SD))	35.5 (10.7)	37.1 (10.1)	41.5 (9.3)	< 0.001
SF-12 MCS (mean (SD))	38.5 (10.9)	40.8 (10.1)	46.9 (8.5)	< 0.001
EQ-Index (mean (SD))	0.691 (0.207)	0.716 (0.196)	0.792 (0.149)	< 0.001
EQ-VAS (mean (SD))	52.9 (22.1)	55.0 (19.4)	63.6 (18.6)	< 0.001
IWQOL-lite Total Score (mean (SD))	41.6 (21.1)	44.9 (20.4)	49.9 (19.3)	< 0.001

^a^Using ANOVA for continuous variables and chi-square for dichotomous variables.

BMI, body mass index; EQ-Index, EQ-5D questionnaire index score; EQ-VAS, EQ-5D questionnaire visual analog scale score; IWQOL-Lite, Impact of Weight on Quality of Life - Lite questionnaire; MCS, mental component summary score; PCS, physical component summary score; SD, standard deviation; SF-12, Short Form 12 questionnaire.

### Follow-up and missing data

At two years, weight and BMI data were 83% complete and HRQL questionnaires were 87% complete for the SF-12 and 89% complete for the EQ-5D and IWQOL-Lite. Overall, 93 (62%) wait-listed patients crossed over to medical management and 50 (25%) medically managed patients crossed-over to surgery. The mean time to transition was, on average, 22 months (SD 4) for the wait-list group and 14 months (SD 7) for the medically treated patients. No wait-listed patients transitioned directly to surgery.

### Weight change at two years

A full description has been published elsewhere [14]. Mean two-year weight losses (SD) were 1.5 (8.5) kg or 0.9 (6.1)% for the wait-list group, 4.1 (11.6) kg or 2.8 (8.0)% for the medical group and 22.0 (19.7) kg or 16.3 (13.6)% for the surgical group (*P* <0.001). At two years, 17%, 32% and 75% of patients lost at least 5% of their initial body weight, and 9%, 17% and 63% lost at least 10% of their initial body weight in the wait-listed, medically managed and surgically treated groups, respectively (*P* <0.001 for all).

### Instrument specific changes in HRQL over two years

**Figure 1:**
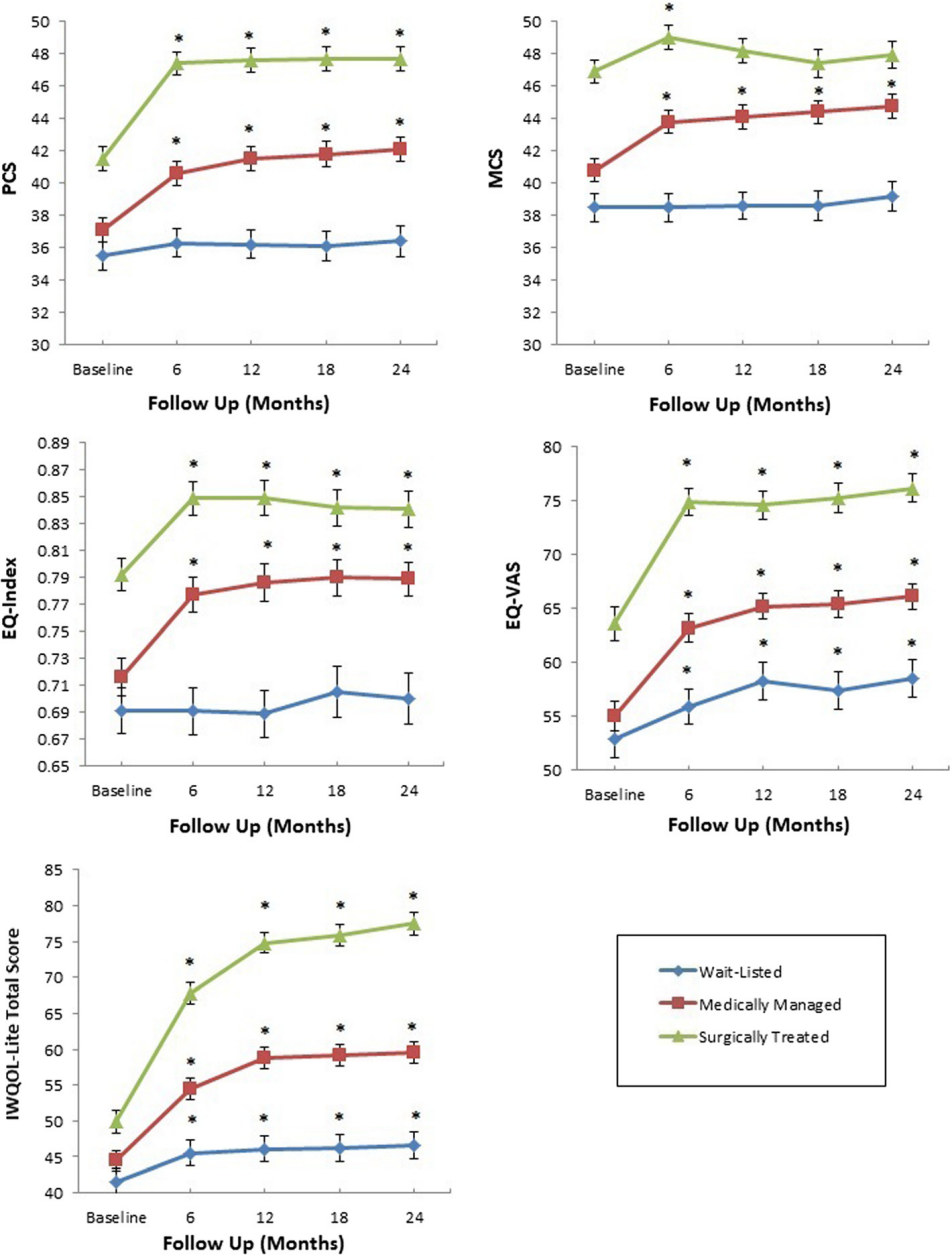
**Health-related quality of life change by study group.** Error bars depict ± standard error. EQ-index, EQ-5D index score; EQ-VAS, EQ-5D visual analog scale; IWQOL-Lite, Impact of Weight on Quality of Life – Lite questionnaire; MCS, Short form-12 mental component summary score; PCS, Short form-12 physical component summary score. *P <0.05 versus baseline.

**Figure 2:**
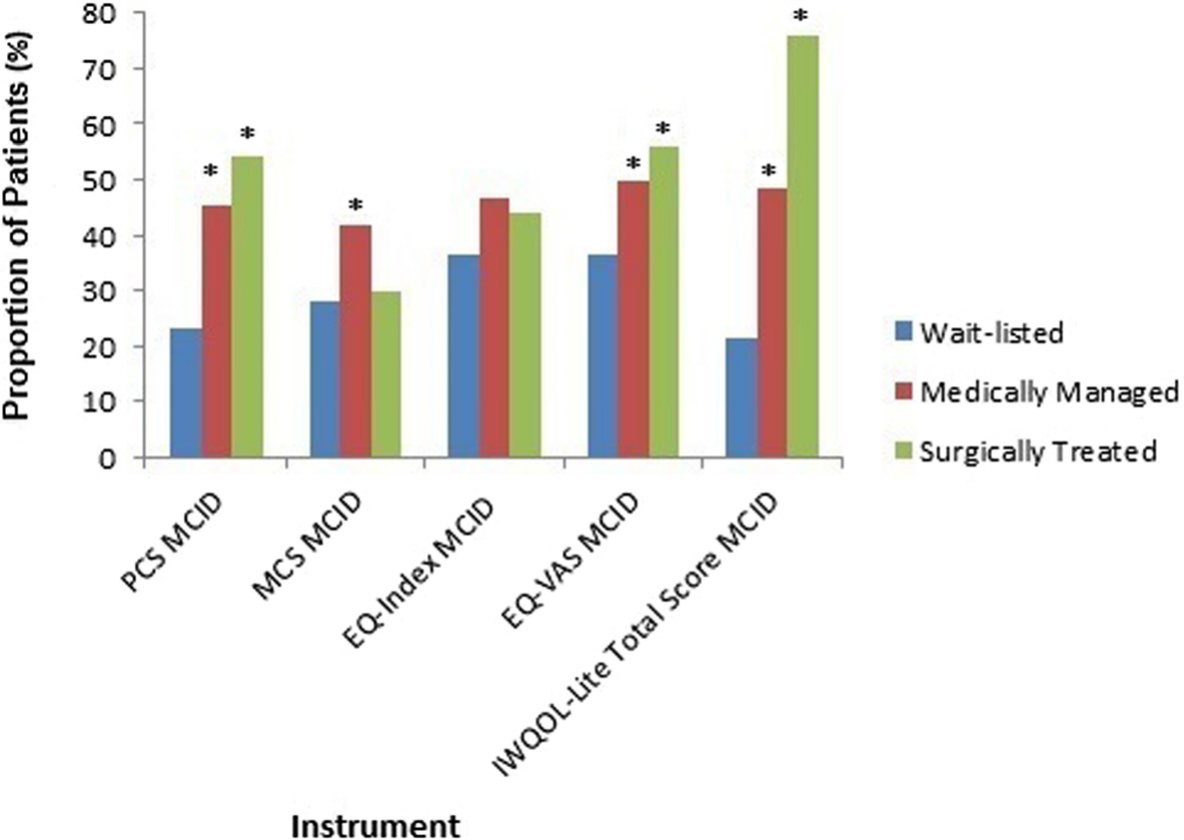
**Proportion of patients achieving MCIDs.** PCS MCID = 5 points; MCS MCID = 5 points, EQ-Index MCID = 0.03 points; EQ-VAS MCID = 10 points; IWQOL-Lite Total Score MCID = 12 points. EQ-index, EQ-5D questionnaire index score; EQ-VAS, EQ-5D visual analog scale; IWQOL-Lite, Impact of Weight on Quality of Life – Lite questionnaire; MCS, Short Form-12 questionnaire mental component summary score; PCS, Short Form-12 questionnaire physical component summary score. *P <0.05 versus wait-listed.

Most improvements in HRQL occurred within six months of study entry (Figure 1). At two years, the mean PCS improved significantly more in the surgical and medical groups compared to the wait-listed group (*P* <0.001 for both comparisons) (Table 2). Surgical patients reported statistically significant (*P* = 0.004), but not clinically important (2.3 points) improvements in PCS score compared to medical patients. For the PCS scores, the five-point MCID was reached in 23% of wait-listed, 46% of medically, and 54% of surgically treated patients (*P* <0.001 for all groups, *P* = 0.12 for the medical versus surgical group) (Figure 2).

**Table 2:** **Two-year changes in HRQL scores**

	**Unadjusted change from baseline (mean (SD))**				**Adjusted difference in differences (points (95% ** **CI))**		
**Instrument**	**Wait-listed**	**Medical management**	**Surgical treatment**	***P*** **-value** ^**a**^	**Medical (wait-list subtracted)**	**Surgery (wait-list subtracted)**	**Surgery (medical subtracted)**
	**(n = 150)**	**(n = 200)**	**(n = 150)**				
SF-12 PCS	1.0 (6.7)	5.0 (7.1)	6.2 (9.2)	<0.001	4.5 (2.9, 6.0)	6.8 (5.1, 8.5)	2.3 (0.7, 3.9)
SF-12 MCS	0.7 (7.7)	4.0 (9.6)	1.0 (10.6)	0.011	4.2 (2.3, 6.0)	3.2 (1.1, 5.3)	−1.0 (−2.9, 0.9)
EQ-Index	0.01 (0.17)	0.07 (0.16)	0.05 (0.18)	0.002	0.07 (0.04, 0.10)	0.08 (0.04, 0.11)	0.00 (−0.03, 0.04)
EQ-VAS	5.7 (19.1)	11.1 (20.8)	12.5 (21.7)	0.009	6.9 (3.4, 10.4)	13.6 (9.7, 17.4)	6.7 (3.1, 10.2)
IWQOL-Lite Total Score	3.2 (13.8)	13.8 (15.5)	26.9 (21.4)	<0.001	11.4 (8.1, 14.7)	25.7 (22.1, 29.3)	14.3 (11.0, 17.6)

^a^ANOVA. Comparisons adjusted for age, sex, baseline BMI and baseline HRQL score. EQ-Index, Euroqol-5D questionnaire index score; EQ-VAS, Euroqol-5D questionnaire visual analog scale score; HRQL, health-related quality of life; IWQOL-Lite, Impact of Weight on Quality of Life - Lite questionnaire; MCS, mental component summary score; PCS, physical component summary score; SF-12, Short Form 12 questionnaire.

The mean MCS improved significantly in surgical and medical groups, compared to the wait-listed group (*P* = 0.003 and *P* <0.001, respectively), with no significant difference between the medical and surgical groups (*P* = 0.32) (Table 2). None of these differences attained MCID thresholds. The five-point MCID was reached for 28% of wait-listed, 42% of medically and 30% of surgically treated patients (*P* = 0.01 for all groups, *P* = 0.02 for medical versus surgical) (Figure 2).

The mean EQ-Index improved to a clinically important extent (Table 2) in the surgical and medical groups compared to the wait-listed group (*P* <0.001 for both comparisons). There was no significant difference between the medical and surgical groups (*P* = 0.85). The 0.03-point MCID was reached in 37% of wait-listed patients, 47% of medically and 44% of surgically treated patients (*P* = 0.17 across all groups, *P* = 0.64 for surgical versus medical) (Figure 2).

The mean EQ-VAS improved in both surgical and medical groups compared to the wait-listed group (*P* <0.001 for both comparisons) and between surgically and medically managed patients (*P* <0.001). However, none of these reached the MCID threshold. The 10-point MCID was reached for 37% of wait-listed patients, 50% of medically and 56% of surgically treated patients (*P* = 0.003 for all groups; *P* = 0.27 for surgical versus medical) (Figure 2).

Mean IWQOL-Lite total score improved in each group, with surgical patients showing the greatest improvement (*P* <0.001 for between-group comparisons; Table 2). For the IWOQL-Lite total score, the 12-point MCID was reached for 21% of wait-listed patients, 49% of medically and 76% of surgically treated patients (*P* <0.001 for all groups; *P* <0.001 for surgical versus medical) (Figure 2).

### Weight loss thresholds to achieve minimal important differences in HRQL

Weight losses required to achieve the HRQL MCIDs for each instrument (Table 3) were 23% (95% CI: 17.5, 32.5) for the PCS, 25% (17.6, 40.2) for the MCS, 9% (6.2, 15.0) for the EQ-Index, 23% (17.3, 36.1) for the EQ-VAS and 17% (14.1, 20.4) for the IWQOL-Lite total score. Full multivariable models are presented in Additional file 1: Tables S1 to S3. No MCID thresholds were reached with ≥5% weight loss. With ≥10% weight loss, only the EQ-index score improvement reached the MCID threshold.

**Table 3:** **Predicted weight loss thresholds required to achieve MCID HRQL scores**

**Instrument**	**Established MCID** ^**a**^	**Relative weight loss required to achieve MCID (% weight loss (95%** **CI))**	**Improvement in HRQL achieved with 5%** **weight loss (points (95%** **CI))**	**Improvement in HRQL achieved with 10%** **weight loss (points (95% ** **CI))**
SF-12 PCS	3 to 5^b^	23% (17.5, 32.5)	1.10 (0.77, 1.43)	2.20 (1.54, 2.86)
SF-12 MCS	3 to 5^b^	25% (17.6, 40.2)	1.02 (0.62, 1.42)	2.04 (1.24, 2.84)
EQ-Index	0.03	9% (6.2, 15.0)	0.017 (0.010, 0.024)	0.034 (0.020, 0.048)
EQ-VAS	10	23% (17.3, 36.1)	2.14 (1.39, 2.89)	4.28 (2.77, 5.79)
IWQOL-Lite Total Score	7 to 12^b^	17% (14.1, 20.4)	3.60 (2.94, 4.25)	7.19 (5.88, 8.50)

^a^Minimum increase in score considered to be clinically important based upon available published literature; ^b^upper value used to estimate minimum weight loss required to achieved MCID. CI, confidence interval; EQ-Index, EQ-5D questionnaire index score; EQ-VAS, EQ-5D questionnaire visual analog scale score; HRQL, health-related quality of life; IWQOL-Lite, Impact of Weight on Quality of Life - Lite questionnaire; MCID, minimal clinically important difference; MCS, mental component summary score; PCS, physical component summary score; SF-12, Short Form 12 questionnaire.

## Discussion

Two major findings are noteworthy from this analysis of 500 patients enrolled in a publicly funded Canadian bariatric care program. First, compared to wait-listed patients who lost little weight over two years, HRQL improved following both medical and surgical treatment, with the most clinically important improvements found with surgery. Second, for most HRQL instruments, the percent weight reductions required to achieve HRQL MCIDs are substantially higher than currently promoted thresholds of 5% to 10% and are more in the order of 20% or greater.

Minimum weight loss thresholds of 5% of initial body weight are commonly cited as sufficient to improve health [5,7]. Regulatory agencies also use a 5% placebo-subtracted weight loss threshold as one requirement for approval of new anti-obesity drugs [26]. Our findings suggest that this 5% threshold is not associated with clinically important improvements in HRQL in most patients. Even 10% weight loss was insufficient for most of the HRQL instruments examined, while 20% weight reductions appeared a more appropriate threshold to achieve clinically important HRQL improvement. A recent paper reported that a 1 kg decrease in weight following a modestly successful (5% weight loss on average) two-year behavioral intervention was associated with statistically significant improvements of 0.25 points in the SF-12 PCS, 0.09 points in the SF-12 MCS, 0.54 points in EQ-VAS and 0.002 in EQ-Index score [25,27]. Except for the MCS, these results are similar to those reported in the present study.

The two-year HRQL changes we observed are comparable to those reported in the Utah Obesity Study, a prospective cohort study that enrolled 308 surgical patients, 253 patients who sought to undergo surgery but did not and 272 population-based controls [28]. After two years of follow up, surgically treated patients reported clinically important improvements in the IWQOL-Lite, PCS and MCS scores [29]. In APPLES, surgical-treatment was associated with the greatest improvements in HRQL (compared to the medically managed and wait-listed groups). HRQL improvements in medically managed patients were surprisingly high given that relatively modest weight losses were observed. We speculate that provision of behavior counselling, relatively frequent contact, and/or greater physical activity may have played a role in improving HRQL independent of weight loss, and further research into weight-independent and -dependent effects is needed [30]. Wait-listed patients experienced no change or small improvements in HRQL over two years despite minimal intervention. This confirms that no substantial deterioration in HRQL over this time occurs in patients awaiting bariatric care, which is relevant because patients trying to access publicly-funded bariatric care often face protracted wait times [31], and we can be assured that at least in terms of HRQL there are no overt harms associated with being wait-listed. In fact, there may be small improvements associated with wait listing or, perhaps, the HRQL changes in the wait-listed group are a product of temporal variation.

The relatively large sample size; inclusion of surgical, medical and wait-listed patients; long follow-up; simultaneous use of three validated HRQL measures; and population-representativeness of the study sample are major strengths of this study. However, there are several limitations. First, the interpretation of our results relies entirely upon the accuracy and validity of the HRQL instruments and established HRQL MCIDs – misidentification of an MCID would result in misspecification of a weight loss threshold. This may account for the discrepancy between the relatively low weight losses needed to attain EQ-5D MCIDs and those needed to attain MCIDs with all other instruments. The 0.03 MCID for the EQ-Index score was not derived from a population of obese patients or patients with chronic disease and, thus, may not appropriately generalize to our study population. In addition, the ceiling effects commonly seen in the three level version of the EQ-5D may make it ill-suited to assess HRQL change with weight loss [30]. While there is much debate over the appropriate ways in which to determine HRQL MCIDs [12], the MCIDs for the instruments we used are well-established and widely accepted, and we conservatively predefined our MCIDs using the higher end of the plausible ranges. Second, censoring was high in the wait-listed and medical groups because of the naturalistic study design in which patients were allowed to sequentially cross over to their next treatment phase. We handled this by using an intent-to-treat framework and LOCF imputation for missing data, as is routinely done in randomized trials of obesity management [32]. We note that study attrition was fairly low (<20%) and LOCF imputation was mostly needed for cross-overs into more intensive treatment (wait-listed transitioned to medical management and medical to surgical treatment). LOCF assumes that any observed changes occurring early in follow-up are maintained over two years. This might result in an overly optimistic assessment of treatment effectiveness. Third, baseline between-group imbalances in weight and HRQL were present (likely because of the sequential nature of the data), and while we adjusted for observed differences some residual confounding is possible. Because of the sequential nature of the data, propensity score matching was not used. Fourth, we did not collect data on the extent to which health behaviors changed and, thus, could not assess whether these contributed to weight-independent improvements in HRQL with medical therapy. Last, our study population was predominately white and female, and all were severely obese, and, therefore, our results may not generalize to a more heterogeneous population or necessarily to those with lesser degrees of obesity; however, our recent meta-analysis of weight loss interventions, including participants with initial BMIs ranging between 25 kg/m^2^ and 55 kg/m^2^, showed little to no improvement in HRQL with modest weight loss [32]. As well, Weight Wise is a publicly funded bariatric program in one region in Canada where all patients have universal healthcare coverage, and it may be that we enrolled more treatment-resistant severely obese patients than those typically seen in other studies conducted in other settings.

## Conclusions

In a severely obese population, only bariatric surgery consistently led to statistically significant and clinically important weight reductions. Medical-management may have led to weight-independent HRQL improvements and this requires further study. HRQL increments per percent of weight loss were small and, for most severely obese patients and most instruments, a 20% weight loss over two years is required to achieve clinically important HRQL improvements predictably. If replicated, these findings also indicate that, from the HRQL perspective, future non-surgical obesity treatments will need to be more efficacious than current ones if clinically meaningful HRQL improvements are to be achieved.

## Additional file

Additional file 1: Table S1. SF-12 Models. **Table S2**: EQ-5D Models. **Table S3**: IWQOL-Lite Model.
