# Supplementary material for: Weight loss required by the severely obese to achieve clinically important differences in health-related quality of life: two-year prospective cohort study
Source: BMC Med. 2014 Oct 15;12:175. doi: 10.1186/s12916-014-0175-5 (PMC4212133; doi:10.1186/s12916-014-0175-5)
Supplement: Additional file 1: Table S1. — SF-12 Models. Table S2: EQ-5D Models. Table S3: IWQOL-Lite Model. [file 12916_2014_175_MOESM1_ESM.docx]

| **Table S1: SF-12 Models** | | |
| --- | --- | --- |
| **Physical Component Summary Score** | | |
| **Adjusted R^2^ = 0.24** | | |
| **Covariate** | **β Coefficient (SE)** | **P-value** |
| Weight Loss (per 1% decrease) | 0.22 (0.03) | < 0.001 |
| Age (per 1 year increase) | -0.03 (0.03) | 0.432 |
| Female | -0.77 (0.98) | 0.433 |
| Baseline BMI (per 1 kg/m^2^ decrease) | 0.11 (0.04) | 0.012 |
| Baseline PCS score (per 1 point increase) | -0.30 (0.03) | < 0.001 |
| Surgical group at baseline (compared to waitlist) | 3.33 (0.98) | 0.001 |
| Medical group at baseline (compared to waitlist) | 4.03 (0.76) | < 0.001 |
| **Mental Component Summary Score** | | |
| **Adjusted R^2^ = 0.21** | | |
| **Covariate** | **β Coefficient (SE)** | **P-value** |
| Weight Loss (per 1% decrease) | 0.20 (0.04) | < 0.001 |
| Age (per 1 year increase) | -0.06 (0.04) | 0.134 |
| Female | -1.98 (1.19) | 0.096 |
| Baseline BMI (per 1 kg/m^2^ decrease) | 0.11 (0.05) | 0.026 |
| Baseline MCS score (per 1 point increase) | -0.38 (0.04) | < 0.001 |
| Surgical group at baseline (compared to waitlist) | -0.01 (1.22) | 0.994 |
| Medical group at baseline (compared to waitlist) | 3.74 (0.92) | < 0.001 |
| Higher score indicates greater health-related quality of life improvement. | | |

**Additional file**

| **Table S2: EQ-5D Models** | | |
| --- | --- | --- |
| **Index Score** | | |
| **Adjusted R^2^ = 0.22** | | |
| **Covariate** | **β Coefficient (SE)** | **P-value** |
| Weight Loss (per 1% decrease) | 0.003 (0.001) | < 0.001 |
| Age (per 1 year increase) | -0.001 (0.001) | 0.176 |
| Female | 0.005 (0.020) | 0.803 |
| Baseline BMI (per 1 kg/m^2^ decrease) | 0.002 (0.001) | 0.061 |
| Baseline Index score (per 1 point increase) | -0.394 (0.037) | < 0.001 |
| Surgical group at baseline (compared to waitlist) | 0.022 (0.021) | 0.3 |
| Medical group at baseline (compared to waitlist) | 0.403 (0.069) | < 0.001 |
| **Visual Analog Scale (VAS)** | | |
| **Adjusted R^2^ = 0.40** | | |
| **Covariate** | **β Coefficient (SE)** | **P-value** |
| Weight Loss (per 1% decrease) | 0.43 (0.08) | < 0.001 |
| Age (per 1 year increase) | -0.05 (0.07) | 0.465 |
| Female | -2.4 (2.24) | 0.284 |
| Baseline BMI (per 1 kg/m^2^ decrease) | 0.12 (0.09) | 0.207 |
| Baseline VAS score (per 1 point increase) | -0.63 (0.04) | < 0.001 |
| Surgical group at baseline (compared to waitlist) | 6.74 (2.27) | 0.003 |
| Medical group at baseline (compared to waitlist) | 6.00 (1.74) | 0.001 |
| Higher score indicates greater health-related quality of life improvement. | | |

| **Table S3: IWQOL-Lite Model** | | |
| --- | --- | --- |
| **Total Score** | | |
| **Adjusted R^2^ = 0.48** | | |
| **Covariate** | **β Coefficient (SE)** | **P-value** |
| Weight Loss (per 1% decrease) | 0.72 (0.07) | < 0.001 |
| Age (per 1 year increase) | -0.06 (0.07) | 0.377 |
| Female | 0.27 (1.95) | 0.892 |
| Baseline BMI (per 1 kg/m^2^ decrease) | 0.41 (0.08) | < 0.001 |
| Baseline Total score (per 1 point increase) | -0.34 (0.03) | < 0.001 |
| Surgical-Treatment (compared to waitlist) | 14.2 (1.95) | < 0.001 |
| Medical-Treatment (compared to waitlist) | 9.89 (1.51) | < 0.001 |
| Higher score indicates greater health-related quality of life improvement. IWQOL, Impact of Weight on Quality of Life. | | |
